# Effects of *APOE*, *APOB* and *LDLR* variants on serum lipids and lack of association with xanthelasma in individuals from Southeastern Brazil

**DOI:** 10.1590/S1415-47572009005000028

**Published:** 2009-06-01

**Authors:** Marcelo A. Nakazone, Miguel A. De Marchi, Marcela A. S. Pinhel, Carolina F. D. C. Barros, Maysa A. F. Júlio, Anielli Pinheiro, Simone S. Arazi, Júlia K. Hotta, Mário H. Hirata, Rosario D. C. Hirata, José E. dos Santos, Dorotéia R. S. Souza

**Affiliations:** 1 Departamento de Biologia Molecular, Faculdade de Medicina de São José do Rio Preto, São José do Rio Preto, SPBrazil; 2 Departamento de Análises Clínicas e Toxicológicas, Faculdade de Ciências Farmacêuticas, Universidade de São Paulo, São Paulo, SPBrazil; 3 Departamento de Clínica Médica, Faculdade de Medicina de Ribeirão Preto, Universidade de São Paulo, Ribeirão Preto, SPBrazil

**Keywords:** apolipoprotein B, apolipoprotein E, LDL receptor, gene polymorphisms, xanthelasma

## Abstract

Xanthelasma might be a clinical manifestation of dyslipidemia, a recognized risk factor for coronary artery disease. We investigated the association of apolipoprotein E (*APOE Hha*I), apolipoprotein B (*APOB Xba*I and *Ins/Del*) and LDL receptor (*LDLR Ava*II and *Hinc*II) gene polymorphisms with lipid profiles in 100 Brazilians with xanthelasma and 100 controls. Allele frequencies were similar in both groups. *APOE*, *APOB* and *LDLR* genotypes were not correlated with differences in the serum lipid profile. In individuals with xanthelasma, the *APOB* D allele was associated with less chance of having increased LDL-cholesterol (O.R. = 0.16, CI95% = 0.03-0.94, p = 0.042). In the control group, the *APOB* X+ allele was associated with less chance of having both increased total cholesterol (O.R. = 0.16, CI95% = 0.03-0.78, p = 0.023) and increased LDL-cholesterol (O.R. = 0.10, CI95% = 0.02-0.60, p = 0.012). Moreover, there was a significantly higher frequency of control individuals (68%) with elevated serum triglyceride levels, compared to patients (48%, p = 0.008). On the other hand, triglyceride levels in controls also seemed to be influenced by all other gene polymorphisms studied, an effect that might be enhanced by environmental factors.

## Introduction

Xanthelasma is the most common form of lipid deposit on the skin, and appears as yellowish plaques on the eyelid or periorbital skin (Bergman, 1994). These have been considered as clinicals sign of both dyslipidemia and advanced atherosclerotic processes, implicated in the pathogenesis of coronary artery disease (CAD) (Ribera *et al.*, 1995), although they have also been found in non-dyslipidemic individuals, thereby suggesting that other factors might be responsible for the formation of these atherogenic manifestations (Tursen *et al.*, 2006).

Variants in the genes encoding apolipoproteins E (*APOE*) and B (*APOB*), as well as the low-density lipoprotein receptor (*LDLR*), have been associated with hypercholesterolemia and the increased risk of CAD in several populations (Hansen *et al.*, 1993; Series *et al.*, 1993; Ribera *et al.*, 1995; Salazar *et al.* 1999; 2000; Cavalli *et al.*, 2000; Pongrapeeporn *et al.*, 2000; Guo *et al.*, 2002; Hirashiki *et al.*, 2003; Rios *et al.*, 2003; Souza *et al.*, 2007). Xanthelasma in association with cardiovascular disease has been reported in individuals with familial hypercholesterolemia and carrying *LDLR* mutations (Norman *et al.*, 1999), and have also been associated with the presence of the APOE*4 allele (Ribera *et al.*, 1995).

With the aim of elucidating the contribution of common polymorphisms from those genes involved in lipid metabolism to the development of xanthelasma, we evaluated whether *APOE* (*Hha*I), *APOB* (*Xba*I and *Ins/Del*) and *LDLR* (*Ava*II and *Hinc*II) gene polymorphisms were associated with the lipid profile encountered in Brazilian individuals with xanthelasma.

## Material and Methods

### Subjects

We investigated 100 unrelated individuals of European descent from southeastern Brazil presenting xanthelasma (37 men and 63 women, between the ages of 32 and 60 years). They were selected by a medical dermatologist in a private dermatology clinic, during the period from April, 2002 to March, 2005. Individuals with liver, renal or thyroid diseases, hypertension, diabetes mellitus, family dyslipidemia, and who had been using lipid-lowering drugs during the prior six months, at least, were precluded. The control group consisted of 100 individuals chosen from the general population (36 men and 64 women, aged from 32 to 57 years), and without any clinical signs of xanthomatosis or any other lipoid metabolic disorder. This study was approved by the Ethics Research Committee of the Faculdade de Medicina de São José do Rio Preto, SP, Brazil (protocol 2277/2002). Furthermore, all the participants signed informative forms, thereby indicating their consent.

### Histopathology

The diagnosis of xanthelasma in all patients was confirmed through histopathological methods. Periorbital skin lesions were surgically excised the tissues being immediately immersed in Carnoy's methanolic fixative (60% methanol, 30% chloroform, and 10% glacial acetic acid) containing 25 μmoles/L of butylated hydroxytoluene and 50 pmoles/L of diethylenetriamine pentaacetic acid, to inhibit any *ex vivo* oxidative modification. The fixed samples were embedded in paraffin according to Sugiyama *et al.* (1992). Xanthelasma histological diagnosis was based on Breier *et al.* (2002) criteria.

### Serum lipid profile

The lipid profile in serum was determined from peripheral blood samples drawn after a 12 hours' fast. The serum concentrations of triglycerides (TG) and total cholesterol (TC) were defined by enzymatic colorimetric methods (Bucolo and David, 1973; Allain *et al.*, 1974). High-density lipoprotein cholesterol (HDL-cholesterol) was determined by precipitation with dextran-magnesium chloride. Low-density lipoprotein (LDL-cholesterol) and very low-density lipoprotein cholesterol (VLDL-cholesterol) were calculated by the Friedewald formula for TG levels below 400 mg/dL (Friedewald *et al.*, 1972). TC/HDL-cholesterol, LDL-cholesterol/HDL-cholesterol and TG/HDL-cholesterol ratios were calculated (Da Luz *et al.*, 2005). Reference values were those recommended by the III Brazilian Guidelines on Dyslipidemias and Guideline of Atherosclerosis Prevention from Atherosclerosis Department of Sociedade Brasileira de Cardiologia (Santos and Sociedade Brasileira de Cardiologia, 2001).

### Genomic DNA analysis

Genomic DNA was extracted from peripheral blood leukocytes. *APOE* [APOE*2 (Cys176Arg, rs7412), APOE*4 (Cys130Arg, rs429358)], *APOB* [*Xba*I (Thr2515Thr, rs693)], *LDLR* [*Ava*II (Val653Val, rs5925) and *Hinc*II (Asn591Asn, rs688)] gene polymorphisms were analyzed through the polymerase chain reaction (PCR) and restriction fragment length polymorphism (RFLP). The *APOB* [*Ins/Del* (rs17240441)] variant was determined by PCR. Amplification was carried out in an Eppendorf-Mastercycler thermal cycler (Eppendorf HQ, Hamburg, Germany). Primers and PCR conditions had been previously described (Hixson and Vernier, 1990; Renges *et al.*, 1991; Boerwinkle *et al.*, 1990; Hobbs *et al.*, 1992). PCR products were analyzed by 1.5% agarose gel electrophoresis after ethidium bromide staining. These were then digested with the endonucleases (Amersham Pharmacia Biotech of Brazil) *Hha*I and *Xba*I for *APOE* and *APOB* single nucleotide polymorphisms (SNPs), respectively, and *Ava*II and *Hinc*II for *LDLR* SNPs, according to manufacturer's instructions. Fragments were identified by electrophoresis on 2% agarose gels (*APOB Xba*I) and 6% polyacrylamide gels (*APOBIns/Del*, *APOEHha*I, *LDLRAva*II and *Hinc*II). Homozygous samples for restriction sites were used as positive control, and 10% of the analyses were repeated randomly.

### Statistical analysis

Categorical variables were compared by chi-squared or Fisher Exact tests. Continuous variables were compared through Mann-Whitney tests. Differences in genotype frequency distribution from Hardy-Weinberg ratios were verified by chi-squared tests. Genotype frequencies were estimated by means of the Expectation-Maximization algorithm using the Arlequin program. Relationships between xanthomatosis and genotypes and other variables were evaluated by univariate logistic regression and multivariate logistic regression analyses with stepwise criteria. The computer package SAS System 6.12 for Windows (SAS Institute Inc., Cary, NC, USA) was used throughout data analyses. Significance was defined as p < 0.05, after application of the Bonferroni correction.

## Results

Biopsy of periorbital skin lesions from all the patients showed a localized collection of histiocytes with a foamy vacuolated cytoplasm, few lymphocytes and neutrophils in the dermis, the absence of fibrosis, and the presence of giant Touton cells (data not shown).

Table 1 shows demographic, biochemical and genotypic data from the study and control groups. Tested variables revealed no diversity between individuals with xanthelasma and controls. Allelic frequencies for *APOE*, *APOB* and *LDLR* polymorphisms were similar in both groups, and all the five polymorphisms exhibited the distribution patterns predicted by Hardy-Weinberg equilibrium (HWE), except *APOBXba*I for patients (x^2^_(1)_ = 3.97; 0.02 < p < 0.05).

No relationships were detected between gene polymorphisms and serum lipids in either of the two groups (Tables 2  and 3). In the control group, an increased TG/HDL-cholesterol ratio was observed in *LDLR* H+H+ genotype carriers (p = 0.036) when compared to non-carriers (data not shown).

Univariate logistic regression analysis showed that individuals with xanthelasma stand less chance of having increased TG (p = 0.014) and VLDL-cholesterol (p = 0.014; Table 4), and that individuals with lower TG (< 150 mg/dL) present an increased risk for xanthelasma (O.R. = 2.30, CI95% = 1.30-4.09, p = 0.005) (data not shown). Nevertheless, no relationships between gene polymorphisms and xanthelasma were found (Table 4).

Multivariate logistic regression analysis indicated that the presence of *APOB* X+ allele was associated with less chance of a rise in TC (≥ 200 mg/dL) (O.R. = 0.16, CI95% = 0.03-0.78, p = 0.023) and LDL-cholesterol (≥ 130 mg/dL) (O.R. = 0.10, CI95% = 0.02-0.60, p = 0.012) in the control group (data not shown). On the other hand, *APOB* D allele was associated with less chance of an increase in LDL-cholesterol (≥ 130 mg/dL) (O.R. = 0.16, CI95% = 0.03-0.94, p = 0.042) in individuals with xanthelasma (data not shown).

## Discussion

In this study, the *APOE*, *APOB* and *LDLR* polymorphisms and serum levels of TC, HDL-cholesterol and LDL-cholesterol were similarly distributed in individuals with xanthelasma without co-morbidities and controls. This reinforces the possibility of local lipid-profile alterations with a deposit of lipids only at dermatological sites (Bergman, 1994; Bergman *et al.*, 1996).

In our series, the allelic and genotypic distributions of *APOEHha*I polymorphism were similar in individuals with xanthelasma and controls, and showed no significant association to lipid profiles in either group. These results are similar to those reported by Gomez *et al.* (1988) and Lin *et al.* (2004). On the other hand, they differ from those by Ribera *et al.* (1995), who detected a significantly increased frequency of the E3E4 genotype in patients with xanthomatosis together with an altered lipid profile. Moreover, the presence of the APOE*2 allele was shown to be a factor related to significantly higher levels of TG and lower levels of TC and LDL-cholesterol in control individuals compared to patients (Eto *et al.*, 1986). This intensifies controversy regarding the protective effect against cardiovascular manifestations conferred by this allele (Yang *et al.*, 2004).

This study showed similarities between the groups when considering allelic frequencies and genotypic combinations of *APOBXba*I and *Ins/Del* gene polymorphisms. The genotypic distribution observed for *APOBXba*I was found to be just marginally different (p = 0.02-0.05) from that expected within the HWE hypothesis in the group of patients.

Multivariate logistic regression analysis with stepwise criteria revealed an association between the presence of the *APOB* X+ allele and less chance of an increase in TC and LDL-cholesterol in the control group, this being contrary to results as reported by Series *et al.* (1993).

Brazilian individuals with xanthelasma, and who were carriers of the *APOB* D allele, presented less chance of undergoing an increase in LDL-cholesterol, this suggesting its association with the lipid profile, as already reported by Hansen *et al.* (1993) in Denmark. However, no associations were detected through other studies (Gaffney *et al.*, 1993; Jemaa *et al.* 2004).

*LDLRAva*II and *Hinc*II polymorphisms did not discriminate groups by their allelic or genotypic distribution, nor did they exert any influence on the lipid profile. Controversial results were reported in Thai subjects by Pongrapeeporn *et al.* (2000), when considering the presence of the A+A+ genotype associated to higher TC and TG, and lower HDL-cholesterol levels. Additionally, Salazar *et al.* (1999) demonstrated increased TC and LDL-cholesterol levels in Brazilian carriers of the H+H+ genotype.

In conclusion, our data show that *apoE*, *apoB* and *LDLR* polymorphisms are not associated with a predisposition for xanthelasma. However, distinct *APOB* polymorphisms are highlighted as risk factors for alterations of the lipid profile in individuals with xanthelasma (*APOB* D) and controls (*APOB* X+).

## Figures and Tables

**Table 1:** Demographic, biochemical and genotypic data of Brazilian individuals with xanthelasma and controls.

Variables	Individuals with Xanthelasma (n = 100)	Controls (n = 100)	Pc
Age (years)	46.2 ± 13.6	44.0 ± 12.6	1.000
Gender (men, %)	37	36	1.000
Total cholesterol (mg/dL)	203.6 ± 26.9	206.1 ± 32.7	1.000
HDL-c (mg/dL)	46.9 ± 8.3	46.4 ± 7.6	1.000
LDL-c (mg/dL)	126.3 ± 26.3	126.3 ± 35.6	1.000
VLDL-c (mg/dL)	30.0 ± 8.4	32.7 ± 6.6	0.080
Triglycerides (mg/dL)	149.8 ± 41.8	163.4 ± 33.0	0.080
TC/HDL-c	4.5 ± 1.0	4.6 ± 1.3	1.000
LDL-c/HDL-c	2.8 ± 0.9	2.9 ± 1.2	1.000
TG/HDL-c	3.3 ± 1.2	3.6 ± 1.0	0.640
*APOEHha*I (%)	(n = 50)	(n = 89)	
APOE*2 allele	7.0	4.0	1.000
APOE*3 allele	84.0	82.0	
APOE*4 allele	9.0	14.0	
*APOBXba*I (%)	(n = 99)	(n = 100)	
X+ allele	40.0	40.0	1.000
X- allele	60.0	60.0	
*APOBIns/Del* (%)	(n = 99)	(n = 100)	
I allele	63.0	58.0	0.800
D allele	37.0	42.0	
*LDLRAva*II (%)	(n = 50)	(n = 50)	
A*+* allele	32.0	43.0	0.288
A- allele	68.0	57.0	
*LDLR Hinc*II (%)	(n = 86)	(n = 100)	
H*+* allele	66.0	60.0	0.522
H- allele	34.0	40.0	

n , number of individuals. TC, total cholesterol; HDL-c, high-density lipoprotein cholesterol; LDL-c, low-density lipoprotein cholesterol; VLDL-c, very low-density lipoprotein cholesterol; TG, triglycerides. +/-, presence/ absence of restriction site. *Ins/Del*, insertion/deletion. Continuous variable data are reported as mean ± SD and compared by the Mann-Whitney test. Categorical variables were compared by chi-squared and the Fisher exact tests. Pc = probability-value in reference to Bonferroni correction.

**Table 2:** Relationships between serum lipids and *APOE*, *APOB* and *LDLR* polymorphisms in individuals with xanthelasma. No statistically significant differences were found.

Polymorphisms		Lipids (mg/dL)				
		TC	HDL-c	LDL-c	VLDL-c	TG
*APOE Hha*I	E2E3 (7)	204.9 ± 40.2	46.3 ± 6.1	131.7 ± 42.4	26.9 ± 4.0	134.3 ± 19.5
	E3E3 (34)	204.8 ± 23.6	46.5 ± 8.0	125.6 ± 26.6	32.6 ± 6.9	162.5 ± 34.4
	E3E4 (9)	204.1 ± 14.0	47.7 ± 10.1	122.9 ± 13.6	33.6 ± 7.2	167.4 ± 36.2
	P	0.422	0.893	0.801	0.073	0.069
	Pc	1.000	1.000	1.000	0.219	0.207
*APOB Xba*I	X+X+ (11)	208.0 ± 22.4	49.1 ± 7.8	126.4 ± 22.0	32.5 ± 6.4	162.6 ± 32.2
	X+X- (57)	203.0 ± 25.4	47.5 ± 7.5	126.3 ± 24.5	28.9 ± 8.7	144.1 ± 43.3
	X-X- (31)	203.8 ± 31.6	45.4 ± 9.5	126.6 ± 31.4	31.2 ± 8.4	155.5 ± 41.9
	P	0.759	0.343	0.915	0.079	0.071
	Pc	1.000	1.000	1.000	0.237	0.213
*APOB Ins/Del*	II (37)	206.1 ± 32.2	47.3 ± 9.7	128.1 ± 30.8	28.7 ± 7.8	143.1 ± 38.8
	ID (50)	202.3 ± 23.4	46.1 ± 7.2	125.7 ± 23.5	31.2 ± 9.1	155.4 ± 45.5
	DD (12)	203.3 ± 24.6	49.7 ± 7.5	124.3 ± 24.9	29.3 ± 6.9	146.3 ± 35.1
	P	0.702	0.461	0.820	0.398	0.432
	Pc	1.000	1.000	1.000	1.000	1.000
*LDLR Ava*II	A+A+ (3)	230.7 ± 54.9	41.7 ± 5.7	156.3 ± 60.1	32.7 ± 5.0	163.0 ± 24.6
	A+A- (26)	203.0 ± 21.9	45.7 ± 8.5	125.5 ± 24.0	31.7 ± 7.9	158.0 ± 39.2
	A-A- (21)	203.1 ± 21.7	48.7 ± 7.5	122.2 ± 23.9	32.2 ± 5.9	160.8 ± 29.4
	P	0.409	0.169	0.403	0.906	0.893
	Pc	1.000	0.507	1.000	1.000	1.000
*LDLRHinc*II	H+H+ (35)	198.3 ± 21.2	47.9 ± 8.1	118.9 ± 20.2	31.5 ± 8.4	157.4 ± 41.9
	H+H- (43)	208.2 ± 27.5	46.5 ± 8.9	131.5 ± 27.3	29.1 ± 7.6	145.3 ± 38.1
	H-H- (8)	207.9 ± 45.7	47.0 ± 7.6	133.6 ± 43.7	27.3 ± 13.5	136.0 ± 67.6
	P	0.276	0.687	0.191	0.713	0.683
	Pc	0.828	1.000	0.573	1.000	1.000

Number of individuals between parenthesis. TC, total cholesterol; HDL-c, high-density lipoprotein cholesterol; LDL-c, low-density lipoprotein cholesterol; VLDL-c, very low-density lipoprotein cholesterol; TG, triglycerides. +/- indicates the presence/absence of a restriction site. *Ins/Del* indicates insertion/deletion. Data are reported as mean±SD and compared by the Mann-Whitney test. Values for *APOE* genotypes were compared by the Kruskal-Wallis test. Pc = probability-value in reference to Bonferroni correction.

**Table 3:** Relationships between serum lipids and *APOE*, *APOB* and *LDLR* polymorphisms in the control group. No statistically significant differences were found.

Polymorphisms		Lipids (mg/dL)				
		TC	HDL-c	LDL-c	VLDL-c	TG
*APOE Hha*I	E2E3 (4)	186.5 ± 9.8	44.3 ± 4.6	108.3 ± 16.4	34.0 ± 2.7	169.3 ± 13.3
	E3E3 (63)	206.5 ± 32.7	47.3 ± 7.5	126.2 ± 35.4	33.0 ± 6.6	164.7 ± 33.1
	E3E4 (16)	213.3 ± 36.3	45.8 ± 8.0	132.3 ± 39.5	35.1 ± 5.5	175.4 ± 27.4
	P	0.261	0.628	0.543	0.142	0.160
	Pc	0.783	1.000	1.000	0.426	0.480
*APOB Xba*I	X+X+ (12)	206.3 ± 36.1	48.4 ± 6.8	122.8 ± 27.5	35.0 ± 11.4	174.2 ± 56.6
	X+X- (56)	201.9 ± 30.1	47.2 ± 7.7	121.3 ± 35.1	32.2 ± 5.8	160.6 ± 29.1
	X-X- (32)	213.4 ± 35.6	44.2 ± 7.4	136.3 ± 38.1	32.9 ± 5.6	164.1 ± 28.1
	P	0.226	0.152	0.159	0.897	0.924
	Pc	0.678	0.456	0.477	1.000	1.000
*APOB Ins/Del*	II (30)	210.0 ± 39.6	45.1 ± 7.6	130.3 ± 45.4	32.1 ± 7.1	160.3 ± 36.0
	ID (56)	206.8 ± 31.9	47.0 ± 7.5	126.1 ± 33.2	33.7 ± 6.6	167.9 ± 32.7
	DD (14)	195.1 ± 13.7	46.6 ± 8.3	118.1 ± 17.3	30.4 ± 4.9	151.9 ± 25.2
	P	0.769	0.614	0.851	0.260	0.300
	Pc	1.000	1.000	1.000	0.780	0.900
*LDLR Ava*II	A+A+ (9)	209.9 ± 29.2	46.6 ± 6.2	131.2 ± 36.0	32.1 ± 3.4	159.9 ± 17.4
	A+A- (25)	211.2 ± 38.6	48.0 ± 7.5	129.2 ± 40.0	34.0 ± 8.9	169.8 ± 44.0
	A-A- (16)	203.3 ± 22.6	45.7 ± 6.9	122.9 ± 25.6	34.6 ± 5.6	173.1 ± 28.4
	P	0.960	0.499	0.943	0.556	0.540
	Pc	1.000	1.000	1.000	1.000	1.000
*LDLRHinc*II	H+H+ (32)	200.9 ± 27.5	44.3 ± 7.2	120.3 ± 33.8	33.8 ± 5.9	168.8 ± 29.8
	H+H- (55)	203.6 ± 31.3	47.9 ± 7.8	124.0 ± 31.9	32.1 ± 7.3	160.2 ± 36.2
	H-H- (13)	229.4 ± 42.4	45.2 ± 7.1	150.3 ± 47.0	32.7 ± 5.1	163.6 ± 26.2
	P	0.095	0.102	0.119	0.265	0.259
	Pc	0.285	0.306	0.357	0.795	0.777

Number of individuals between parentheses. TC, total cholesterol; HDL-c, high-density lipoprotein cholesterol; LDL-c, low-density lipoprotein cholesterol; VLDL-c, very low-density lipoprotein cholesterol; TG, triglycerides. +/- indicates the presence/absence of a restriction site. *Ins/Del* indicates insertion/deletion. Data are reported as mean±SD and compared by the Mann-Whitney test. Values for *APOE* genotypes were compared by the Kruskal-Wallis test. Pc, probabilities in reference to the Bonferroni correction.

**Table 4:** Univariate logistic regression analysis of the variables associated with xanthelasma.

Variables	Category	p-value	Odds ratio	95% confidence interval
Age (years)	Continuous	0.225	1.01	0.99-1.04
Gender	Male	0.883	1.04	0.59-1.86
*APOEHha*I	APOE*2 allele	0.255	1.91	0.63-5.79
	APOE*3 allele	0.129	7.86	0.43-142.55
	APOE*4 allele	0.363	0.67	0.28-1.59
*APOBXba*I	X+ allele	0.917	1.03	0.57-1.88
	X- allele	0.845	1.09	0.46-2.60
*APOBIns/Del*	I allele	0.694	1.18	0.52-2.70
	D allele	0.272	0.72	0.40-1.30
*LDLRAva*II	A+ allele	0.302	0.65	0.29-1.47
	A- allele	0.078	3.44	0.87-13.56
*LDLRHinc*II	H+ allele	0.429	1.46	0.57-3.70
	H- allele	0.219	0.69	0.38-1.25
Total cholesterol (mg/dL)	Continuous	0.560	0.99	0.99-1.01
LDL-c (mg/dL)	Continuous	0.991	1.00	0.99-1.01
HDL-c (mg/dL)	Continuous	0.655	1.01	0.97-1.04
VLDL-c (mg/dL)	Continuous	0.014^#^	0.95	0.91-0.99
Triglycerides (mg/dL)	Continuous	0.014^#^	0.99	0.98-0.99
TC/HDL-c	Continuous	0.426	0.91	0.72-1.15
LDL-c/HDL-c	Continuous	0.691	0.95	0.73-1.24
TG/HDL-c	Continuous	0.049	0.77	0.59-0.99

+/- = presence/absence of a restriction site. *Ins/Del*, insertion/deletion. HDL-c, high-density lipoprotein cholesterol; LDL-c, low-density lipoprotein cholesterol; VLDL-c, very low-density lipoprotein cholesterol; TC, total cholesterol; TG, triglycerides. ^#^Statistically significant difference considering variable associated to xanthelasma.
